# Mechanisms of Esophago-Pharyngeal Acid Regurgitation in Human Subjects

**DOI:** 10.1371/journal.pone.0022630

**Published:** 2011-07-22

**Authors:** Michal Marcin Szczesniak, Rohan Benjamin Williams, Ian James Cook

**Affiliations:** Department of Gastroenterology, University of New South Wales and St George Hospital, Sydney, New South Wales, Australia; Vanderbilt University Medical Center, United States of America

## Abstract

Esophago-pharyngeal regurgitation is implicated in various otolaryngologic and respiratory disorders. The pathophysiological mechanisms causing regurgitation are still largely unknown.

**Aim:**

To determine the principal mechanisms behind esophago-pharyngeal regurgitation.

**Methods:**

We studied 11 patients with extra-esophageal GORD symptoms in whom esophago-pharyngeal acid regurgitation had previously been demonstrated using ambulatory, dual (pharyngo-esophageal) pH metry (>2 episodes/day using previously validated pH-metric criteria). Patients underwent continuous, 24 hr, stationary monitoring of pharyngo-esophageal manometry and dual (pharyngeal and esophageal) pH recordings. They were intubated with a 14-channel manometric assembly incorporating 2 sleeve sensors monitoring the upper and lower esophageal sphincters simultaneously. A dual pH catheter recorded pH signals 2 cm above the UES midpoint and 7 cm above the LES midpoint.

**Results:**

A total of 32 episodes of spontaneous esophago-pharyngeal acid regurgitation were recorded. All episodes occurred in the upright posture and 91% occurred within 3 hrs post-prandium. All regurgitation events were associated with a relaxation of the UES, which were classified as transient non-swallow related relaxations in 29 (91%) and swallow-related in the remaining 3 (9%). Straining was an additional associated factor in 41% of regurgitation events, but strain alone was not sufficient to cause esophago-pharyngeal regurgitation.

**Conclusion:**

Some form of active UES relaxation is necessary for regurgitation to occur. The dominant mechanism underlying esophago-pharyngeal acid regurgitation is the non-swallow related, transient UES relaxation.

**Level of Evidence:**

N/A

## Introduction

Esophago-pharyngeal regurgitation is implicated in otolaryngologic and respiratory disorders such as cough, asthma and laryngitis [1]–[3]. However, categorical proof of a causative link between pH events and symptoms in these disorders in individual patients remains elusive. The pathophysiological mechanisms causing esophago-pharyngeal regurgitation, that can potentially lead to injury of supra-esophageal structures, are largely unknown. Factors leading to the development of gastro-esophageal reflux disease may not play a central role, as a large proportion of patients with laryngitis that is believed to be acid-related, have normal esophageal motility and physiological levels of esophageal acid exposure [2], [4], [5]. Low basal UES pressure is an unlikely cause, as in healthy individuals UES tone falls to very low levels during sleep without causing regurgitation [6] and UES hypotonia following cricopharyngeal myotomy, even in established refluxers, does not predispose to regurgitation [7]. Previous studies in humans and animals have shown that experimental rapid esophageal distension by gas can trigger the UES relaxation response [8], [9]. Studies by Willing et al. [10] and Torrico et al. [11] measuring UES responses to spontaneous gastro-esophageal reflux events found, that resting UES pressure increased after both acidic and non-acidic reflux events and that these events were associated with esophageal common-cavity episodes. The latter study also reported that 54% of common cavity episodes caused a transient relaxation of the upper esophageal sphincter, a phenomenon akin to that observed during the normal belch reflex [8]. Hence, we hypothesized that the predominant mechanism of esophago-pharyngeal regurgitation is a transient non-swallow related UES relaxation. The aim of the present study was to determine the principal mechanisms behind esophago-pharyngeal regurgitation, by measuring pressure in both esophageal sphincters, within the esophagus and the pharynx simultaneous with pharyngeal and esophageal pH signals.

## Methods

### Patients

Patients were considered for the study if they presented with one or more extra-esophageal symptoms of reflux that indicated the potential for esophago-pharyngeal regurgitation. These patients underwent 24 hr ambulatory dual pH monitoring to quantify the occurrence, if any, of esophago-pharyngeal acid regurgitation, according to previously validated criteria [12]. All patients who showed more than one regurgitation event were recruited into the study and underwent a prolonged combined manometric and dual pH study (see below).

### Ethics Statement

All subjects gave written informed consent before commencement of the study. The protocol, patient information and consent form were approved by South East Health Human Research Ethics Committees Southern Section.

### Experimental protocol and measurement techniques

**Figure 1:**
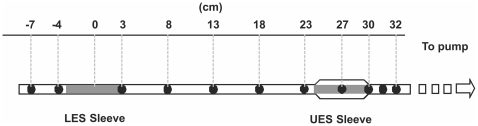
The manometric assembly.

Upper and lower esophageal sphincter pressures and esophageal body pressures were monitored using a custom made silicone manometric assembly (Dentsleeve, Bowden, South Australia). The assembly (3.5 mm outer diameter) had 14 recording lumina (0.4 mm inner diameter) and incorporated a 6 cm sleeve sensor for LES pressure recording and a second 6 cm sleeve sensor with an oval cross section to ensure the antero-posterior orientation of the sleeve in the UES. With the zero position on the catheter being the midpoint of the distal LES sleeve, 12 side holes were spaced along the catheter at −7, −4, 3, 8, 13, 18, 23, 27, 30, 31.5, 32 cm. The UES sleeve midpoint was at 27 cm (Fig. 1). All manometric lumina were perfused by a low-compliance pneumohydrolic perfusion pump at a rate of 0.6 ml/min. Pressures were registered by external transducers (Abbott Critical Care Systems, Singapore).

The manometric assembly was passed transnasally and the proximal sleeve was positioned by station pull-through across the UES. Due to the fixed distance between UES and LES sleeve sensors (27 cm), priority was given for the correct positioning of the UES sleeve. To minimize irritation to the pharynx by the perfusate, all channels above the LES sleeve sensor were not continuously perfused. Pharyngeal channels at the top margin of the UES sleeve were transiently perfused throughout the study to record the baseline pharyngeal pressure. After positioning of the manometry catheter, a dual pH catheter (model 91–0021; Synetics Medical AB, Stockholm, Sweden) with an appropriate inter-electrode distance was selected and positioned such that the proximal electrode was 2 cm above the midpoint of manometrically determined UES and the distal sensor 7 cm above the midpoint of LES. In 8 patients the Synetics Polygraph amplifier (modified for Macintosh by Dr C H Malbert, INRA, France) connected to a Macintosh Quadra950 via a National Instruments data acquisition card. The data acquisition software was written by RBH Williams, based on MAD3 by Dr C H Malbert. Sampling rate was 10 Hz. In the remaining 3 patients, the pH and manometric measurements were simultaneously, amplified, digitised by the MP100 system and recorded at 50 Hz on an Apple Macintosh computer with AcqKowledge software (Biopac, Santa Barbara).

For the duration of the study patients were seated in an upright or semi-recumbent position during daytime and were supine at night. Three standard meals were provided but ingestion of alcohol, tea, coffee and acidic drinks were restricted. Subjects were instructed to note meals, periods in supine position and occurrence of symptoms.

### Data Analysis

#### Measurements and definitions

**Figure 2:**
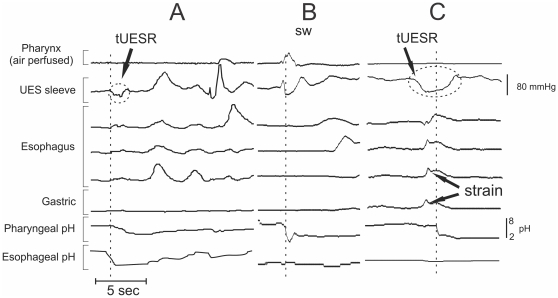
Examples of esophago-pharyngeal regurgitation events. A) Transient UES relaxation (tUESR) occurs shortly after gastro-esophageal reflux, resulting in a rapid fall of pharyngeal pH. Acid is cleared from the pharynx by a swallow; B) Acid regurgitation associated with a swallow occurring during a period of prolonged esophageal acid exposure; C) Strain-related pharyngeal acid regurgitation seen to occur shortly after a preceding UESR – both occurring during a period of prolonged esophageal acid exposure (right).

Initially all episodes of esophago-pharyngeal regurgitation were identified according to criteria defined by Williams et al. [12]. In summary, for a pharyngeal pH drop to be classified as a valid acid regurgitation event it was required to satisfy the following criteria: 1) decrease in pH of at least 2.0 units; 2) nadir pH of <4.0; 3) time from onset of pH decrease to nadir pH, <30 s; 4) pharyngeal pH decrease occurring during a period of esophageal acidification. Examples of esophageal regurgitation events are shown in figure 2. Subsequently the following variables associated with each esophago-pharyngeal regurgitation event were assessed:

### Temporal measures

Time difference between pharyngeal swallow upstroke, if present, and onset of the UES relaxation. The following measure was used to define swallow-related (swUESR) and non-swallow related transient UESR (tUESR).
Delay from onset of gastro-esophageal reflux event to onset of subsequent esophago-pharyngeal regurgitation event. Time difference between onset of pharyngeal pH drop and onset of period of esophageal acidification or a common-cavity associated with the pharyngeal pH drop. In the context of prolonged esophageal acidification, a second reflux event preceding osophago-pharyngeal regurgitation could be masked by a low pH in the esophagus and does not yield a significant change in pH to be classified as yet another reflux event. To circumvent this we also included common-cavity as a marker for reflux if the esophageal pH was <4.

### Manometric measures

Baseline UES pressure - pressure within one sampling interval prior to the onset of UES relaxation.
Nadir UES pressure, lowest pressure during UES relaxation.

### Definitions

Pharyngeal baseline pressure - defined as mean pharyngeal pressure during a 5 s period measured transiently throughout the studies, all UES pressures were referenced to this pressure.
UES hypotonia- defined arbitrarily as UES pressure of less than 15 mmHg during in an interval of demonstrable regurgitation.
Complete UES relaxation - defined as a transient fall in UES tone with the nadir pressure <13 mmHg which is the upper limit of normal for deglutitive UES relaxation with a 2 ml water bolus [13].
Swallow related relaxation (swUESR) - defined as a complete UES relaxation with a pharyngeal swallow upstroke occurring in a time frame consistent with normal range for a swallow. According to previous studies with swallows of 2 ml liquid bolus normal time difference between onset of UESR and onset of mid-pharyngeal closure (onset of major pressure upstroke) is − 0.008 to −0.47 s [14].
Transient UES relaxation (tUESR) - defined as a complete UES relaxation not temporally associated with a pharyngeal swallow upstroke (see above).
Strain - a synchronous and sharp pressure increase >5 mmHg in esophageal and gastric channels.
Common-cavity - a synchronous and abrupt pressure increase > 3 mmHg in at least 3 esophageal channels but not in sphincters nor gastric channels. To differentiate common-cavity phenomenon from respiratory oscillations, the intraluminal pressure was required to reach maximum pressure and plateau in <0.7 s from onset. Normal range for time taken by respiratory oscillations reach maximum amplitude was 0.7–1.4 s.

## Results

Of all the patients referred to our lab for study for the evaluation of presumptive extra-esophageal reflux symptoms over a 12 month period, only eleven patients (6 males, mean age 54 years) of them demonstrated esophago-pharyngeal acid regurgitation during an ambulatory dual sensor pH monitoring. Common presenting problems were: cough, laryngitis, reflux, dysphonia, asthma, globus and recurrent pneumonia (Table 1). Of these 11 with demonstrable ambulatory regurgitation, eight completed full 24 hr static dual pH and pharyngo-esophageal manometry studies and three patients completed a truncated 8–10 hr study due to very limited acute hospital bed availability in the hospital.

**Table 1:** Patient demographics, ambulatory dual pH monitoring results and presenting clinical problems.

Patient	Sex	Age	Ambulatory dual pH		Symptoms and clinical findings
			% T pH<4 (esophagus)	Regurgitation Events(pharyngeal pH drops)	
1	M	48	7.1	2	Post nasal drip; Globus
2	M	71	13.3	16	Cough Dysphonia; Reflux; Sleep apnoea; Hiatus Hernia
3	F	44	1.4	3	Cough; Globus; Dysphonia
4	M	79	49	1	Recurrent pneumonia; Reflux
5	M	64	67.2	8	Pneumonia; Reflux
6	M	59	3.7	3	Intermittent stridor; Dental enamel loss; Laryngitis
7	F	57	1.4	4	Cough; Laryngitis; Asthma
8	F	67	2.5	4	Cough; Laryngitis
9	F	40	19.8	8	Reflux; Scleroderma
10	M	34	26.8	11	Reflux; Laryngitis; Dental erosion
11	F	34	3.6	3	Hoarseness; Cough; Asthma

### Regurgitation Events

Only 4 of 11 ambulant regurgitators demonstrated esophago-pharyngeal regurgitation events during subsequent static combined manometric and pH recording. UES monitoring was achieved in all cases. However, the inter-sphincteric length in these 4 patients was less than 27 cm meaning that LES sleeve sensor was located in the stomach rather than within the LES. A point sensor proximal to the sleeve does not always reliable provide prolonged recording of the LES tone nor transient LES relaxation. A true LES relaxation cannot always reliably be inferred from an observed pressure drop detected by a point sensor in isolation because an axial movement of the sphincter relative to the sensor out of the high pressure zone will register as a similar drop in pressure [15].

**Figure 3:**
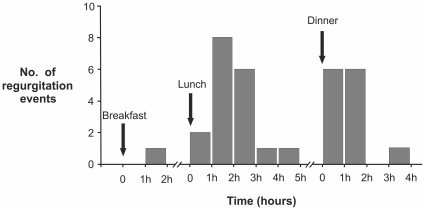
Frequency of regurgitation events after meals. Note that 91% of regurgitation events were recorded within 3 hrs of a meal.

A total of 32 esophago-pharyngeal regurgitation events were recorded and all occurred in the upright posture and 91% occurred within 3 hours post prandium (Fig. 3). The majority of regurgitation events were recorded after lunch and dinner, however we only recorded post breakfast data from two patients who regurgitated because the remaining two subjects underwent truncated day study that did not involve breakfast.

**Figure 4:**
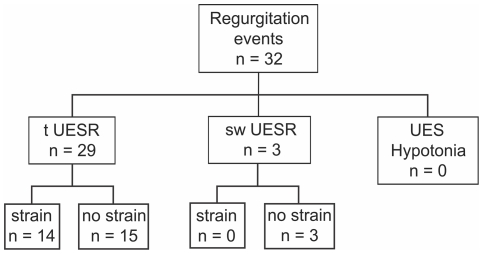
Relationships among esophago-pharyngeal, UES relaxations and strain. The majority of regurgitation events occurred during tUESRs. Abdominal strain can be a factor in regurgitation, but only in the context of a pre-existing UES relaxation. UES hypotonia alone is not an apparent risk factor for regurgitation.

All of the recorded esophago-pharyngeal regurgitation events were associated with a relaxation of the upper esophageal sphincter. These relaxations were classified into three groups according to the criteria defined above. The predominant relaxation type was the transient (non-swallow related) UES relaxation (tUESR) accounting for 29 of 32 (91%) regurgitation events. The remaining 3 (9%) events were associated with swallow-related relaxation of the UES seen to occur during a period of esophageal acidification. None of the recorded regurgitation events occurred during UES hypotonia. Fourteen (43%) events were associated with a strain pattern; all of these being associated with tUESRs (Fig. 4). The esophageal common-cavity phenomenon was synchronous with 12 of 29 (41%) tUESR-related regurgitation events.

**Figure 5:**
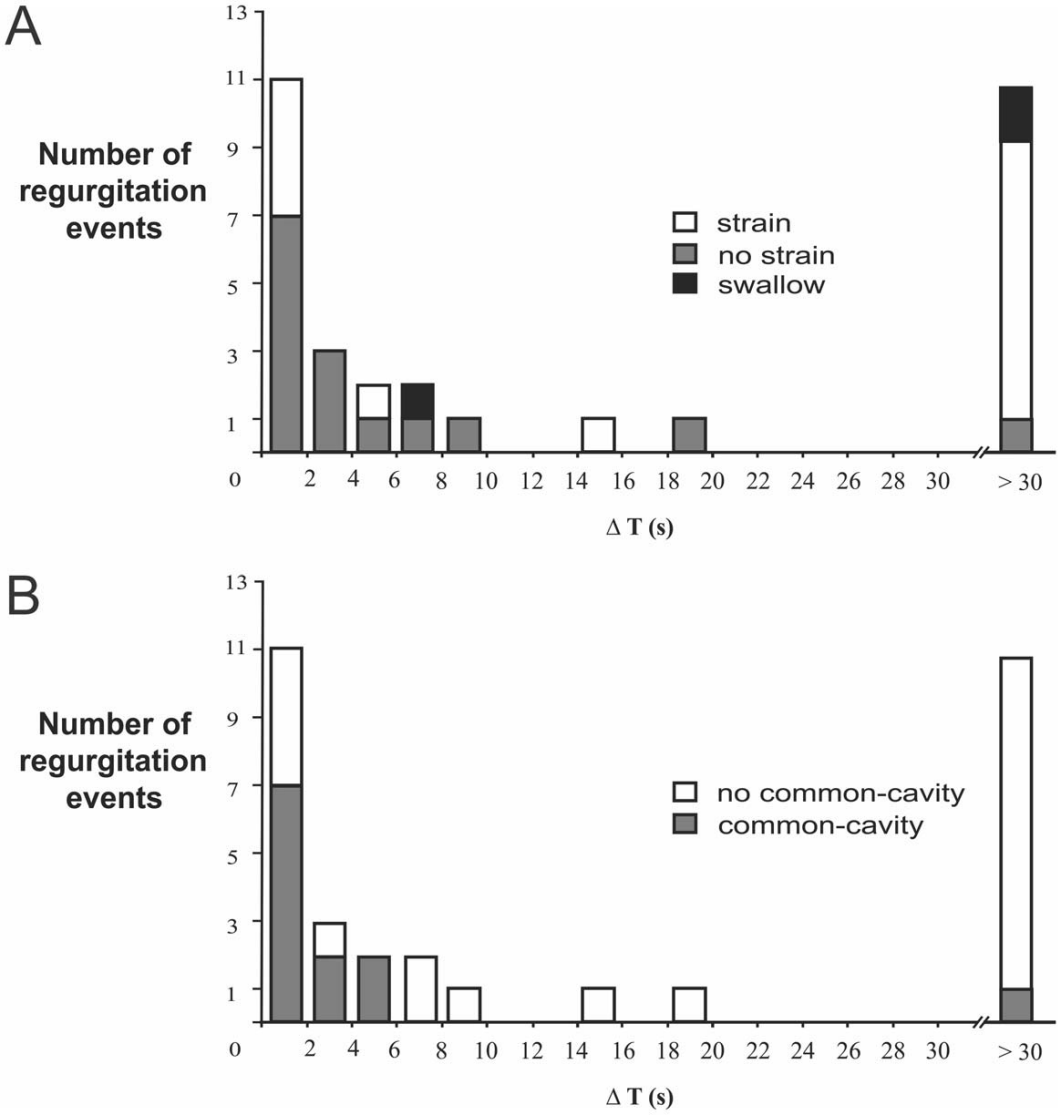
Frequency distribution of all esophago-pharyngeal regurgitation events according to time delay between onset of gastro-esophageal reflux and onset of pharyngeal regurgitation (ΔT). Stratified according to presence of strain (A) and common-cavity (B).

We measured the time delay between onset of gastro-esophageal reflux and subsequent esophago-pharyngeal regurgitation defined as the time lapsed between the onset of esophageal pH drop or a common-cavity and the onset of the pharyngeal pH drop. Sixty percent of all the regurgitation events occurred rapidly within 10 s of a gastro-esophageal reflux event and the remaining events were spaced equally in time as long as 11 min after the antecedant esophageal pH drop. The strain pattern was seen in relation to both immediate (<2 s) and delayed regurgitation events (>30 s). However, the strain pattern was significantly more likely to occur in relation to the delayed events (χ^2^, p<0.05) (Fig 5). Hence, strain seems to facilitate regurgitation but only in the context of a pre-existing UES relaxation.

## Discussion

This study is notable in that it has been able to characterize the mechanisms underlying esophago-pharyngeal regurgitation - a relatively rare event physiological event which may have significant pathophysiological significance. The major findings of this study were: a) all of the regurgitation events occurred in the upright posture; b) some form of UES relaxation is a necessary precursor of esophago-pharyngeal regurgitation; c) the dominant mechanism by which regurgitation occurs is via the transient, non-swallow related UES relaxation (tUESR); c) UES hypotonia is not implicated in regurgitation; d) abdominal strain can facilitate regurgitation but only in the context of a UES relaxation.

The observations in this and previous studies that abrupt esophageal distension, observed as a common-cavity pressure event, precedes a significant proportion of UES relaxations [10] and that experimental air insufflation is a potent stimulus for UES relaxation [9], [16], suggest that abrupt esophageal pressurization (common cavity) mediates the majority of rapid gastro-esophago-pharyngeal events. The available evidence suggests that this mechanism is a vago-vagal reflex [9]. Abdominal strain provides an additional, contributory mechanism for regurgitation in the context of prolonged esophageal acid exposure where strain leads to regurgitation of esophageal contents. However, this only occurs in the context of an already relaxed UES.

Reflux disease is linked to, but is not indicative of supra-esophageal complications of reflux[2], [17]–[20] and even asymptomatic individuals exhibit occasional gastro-esophageal reflux events. This suggests the existence of a mechanism protecting the airways from gastro-esophageal reflux. One of these purported mechanisms is the esophago-UES contractile reflex. Previous studies have shown that common-cavity during reflux events triggered abrupt increase in UES pressure [10], [11]. Our findings showed that 12 of 29 (41%) tUESRs were associated with common-cavities, indicating a failure of the esophago-UES contractile reflex and/or an inappropriate triggering of the UES-relaxation reflex in patients with demonstrated esophago-pharyngeal regurgitation. A failure of the esophago-UES contractile reflex in these patients and/or activation of belch-like response could allow gastric contents to breach the barrier posed by the UES. This mechanism is supported by our study in patients with suspected reflux laryngitis in whom the threshold for esophageal distension-induced UES relaxation is reduced when compared with controls [21].

During screening with 24 hr ambulatory dual pH monitoring of potential candidates for this study, we recorded a total of 63 events in 11 patients (Table 1). The number of regurgitation events recorded in the non-ambulant combined manometry and pH study, was markedly lower than in those 11 participants, only 32 regurgitation events were recorded in 4 individuals. The 50% reduction in frequency of esophago-pharyngeal regurgitation is likely to be due to the sedentary nature of this study. Exercise was shown to induce a threefold increase in esophageal acid exposure in both health and GORD [22], it is likely that performance of normal daily activities has a similar effect on the frequency of esophago-pharyngeal regurgitation when compared to non-ambulant subjects.

A potential shortcoming of this study was the inability to record LES motor events in all patients due to a fixed distance between the sleeve sensors on the manometric assembly and a variable distance between the upper and the lower esophageal sphincters among the subjects. Recently available high resolution manometry catheters with 36 or more sensors circumvent this by enable pressure recording from entire esophagus including both sphincters. Additionally, coupling of the manometry assembly with impedance and pH probe will enable precisely determination the proximal extent of reflux as well as recording both acidic and non-acidic reflux and distinguishing between air, liquid and mixed events in both the pharynx and the esophagus. Another potential weakness of the study is the limited sample size due to challenging nature of such studies and relative rarity of regurgitation events, however observations are compelling and warrant further systematic evaluation in a larger prospective study, preferably coupled with intraluminal impedance recording.

### Conclusion

An active UES relaxation is necessary for esophago-pharyngeal acid regurgitation to occur. The non-swallow related, transient UES relaxation is the dominant mechanism underlying acid regurgitation. Abdominal strain can facilitate regurgitation but only in the context of a concurrent UES relaxation. Hypotonia of the UOS is not implicated in regurgitation.
